# Gender Differences of Health Behaviors in the Risk of Metabolic Syndrome for Middle-Aged Adults: A National Cross-Sectional Study in South Korea

**DOI:** 10.3390/ijerph18073699

**Published:** 2021-04-01

**Authors:** Jaehee Yoon, Jeewuan Kim, Heesook Son

**Affiliations:** 1 Wolchon Elementary School, Seoul 07980, Korea; rnyjh@snu.ac.kr; 2 Department of Statistics and Data Science, Yonsei University, Seoul 03722, Korea; jeewuan@yonsei.ac.kr; 3 Red Cross College of Nursing, Chung-Ang University, Seoul 06974, Korea

**Keywords:** gender differences, metabolic syndrome, health practice index, middle-aged

## Abstract

This study examined gender differences in health behaviors for the risk of metabolic syndrome (MetS) among middle-aged adults using nationally representative data from the Seventh Korea National Health and Nutrition Examination Survey (2016–2018). The sample included data from 8677 middle-aged adults. The Health Practice Index measured health behaviors, including smoking, alcohol use, physical activity, sleeping, eating breakfast, working hours, nutritionally balanced diet, and mental stress. Complex sample multiple logistic regression analyses were conducted to determine the association between the Health Practice Index (HPI) and MetS. Men and women with poor or moderate HPI scores had significantly higher risks of having MetS than those with good HPI scores. Controlling for covariates, high-risk alcohol use (*p* < 0.001) and physical activity (*p* = 0.008) were associated with the risk of MetS in men and women, respectively. Men reporting alcohol use and women lacking a healthy diet were, respectively, 2.056 times (adjusted odds ratio (OR) = 2.056, 95% CI: 1.681–2.514) and 1.306 times (adjusted OR = 1.306, 95% CI: 1.075–1.587) more likely to have increased risks of MetS. Given these gender differences in health behaviors, developing tailored interventions could be beneficial in preventing MetS among middle-aged men and women.

## 1. Introduction

As a risk factor for various chronic diseases, including cardiovascular diseases, type 2 diabetes mellitus [1,2,3,4], myocardial infarction, stroke, and cancer [2,3], metabolic syndrome (MetS) is estimated to affect a quarter of the world’s population [3,5]. In 2018, the prevalence of MetS was 34.3% and 30.5% in American and Korean adults, respectively [6,7]. Further, middle-age is an important period for the prevention and management of MetS owing to the increased susceptibility to MetS during this life stage [8]. In addition, the risk of cardiovascular disease is notably increased in middle-aged adults with MetS [9]. The prevalence of MetS in older adults has been reported to be as high as 50–60% [6,7]; therefore, active national management is required for middle-aged adults to reduce the subsequent development of MetS.

Lifestyle improvement interventions have been primarily used to prevent MetS, promote the health of individuals with MetS, and reduce the risk for cardiovascular diseases [4,9,10,11,12]. Previous studies have indicated that lifestyle interventions can prevent MetS or improve the status of each diagnostic element of MetS [12,13,14,15,16,17].

The Health Practice Index (HPI), first introduced by Breslow et al. in 1965 [4], has been used to identify the individual habits from the numerous lifestyle habits that contribute to the development of diseases. The main lifestyle habits associated with MetS that have been previously identified include diet [18,19], physical activity [3,5,9,20,21], sleeping hours [22], stress [23], smoking [21,23], and alcohol use [23], which are currently included on the HPI. The HPI is beneficial because it narrows the range of individual lifestyle habits that are important to address when preventing and managing MetS. The HPI has the advantage of being able to assess an individual’s overall health-related lifestyle using a total score; however, few studies have applied the HPI in the analysis of MetS, which is closely associated with lifestyle habits, and their findings have been inconsistent regarding the HPI components that are related to MetS. For example, the HPI total score and the components of drinking alcohol and smoking were found to be related to the risk of MetS, but no associations were found with the remaining indicators [4]. Therefore, research that examines the utility of the HPI in the prevention and management of MetS and reevaluates the association between MetS and the HPI is necessary.

It is critical to consider gender to deliver effective interventions for the prevention and management of MetS [10,24,25] since the risk and each diagnostic component of MetS varies by gender [6,7,10,24,26]; additionally, the frequency of the lifestyle habits associated with MetS also differs by gender [21,25,27]. Furthermore, there may be gender differences in the same lifestyle habit associated with MetS. For example, healthy dietary patterns have been found to have a greater protective effect for MetS among women than men [19]. Therefore, research is needed that identifies the differences in lifestyle habits related to MetS by gender and delivers interventions tailored to reflect those gender differences.

Using the HPI framework, the purpose of this study was to examine the associations between MetS and lifestyle habits by gender in a sample of middle-aged adults. In the current study, we tested (1) the presence of gender differences in the lifestyle habits for the risk of MetS and (2) the HPI factors that are associated with the risk of MetS in men and women.

## 2. Materials and Methods

**Figure 1:**
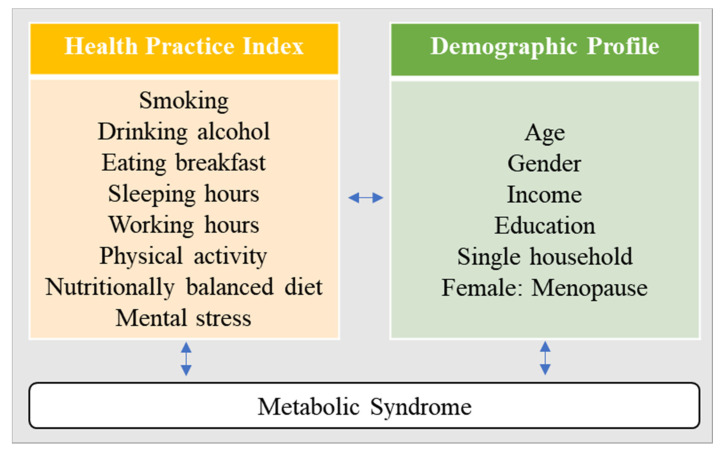
Conceptual framework.

A secondary analysis design was utilized to examine gender differences in health behaviors using the HPI to measure the risk of MetS in a sample of middle-aged men and women. Figure 1 depicts the conceptual framework of the current study.

As a secondary analysis, data from the seventh wave (2016–2018) of the nationally representative Korea National Health and Nutrition Examination Survey (KNHANES) were used. Of the 24,269 individuals included in the dataset, 8677 middle-aged adults (ages 40–64 years) were used in the current study.

### 2.1. Measures

#### 2.1.1. Health Behaviors

To measure health behaviors, we used Morimoto’s HPI version, which was a modification of Breslow’s HPI to reflect Japanese lifestyle [4,28]. A previous study indicated that Morimoto’s HPI was more useful when examining the association between health behaviors and the risk for MetS in Japanese samples compared with Breslow’s HPI [4]. Since there are many similarities in lifestyles between Korea and Japan, which are both Asian cultures, we used Morimoto’s HPI [29].

Morimoto’s HPI consists of eight indicators of health behaviors, including smoking, alcohol use, physical activity, amount of sleep, frequency of eating breakfast, working hours, having a nutritionally balanced diet, and mental stress. Each indicator on the HPI was rated using 1 point, and the total scores were classified into three groups: Poor: 0–3 points; Moderate: 4–5 points; and Good: 6–8 points.

In previous research, the operationalized definitions of each indicator have differed slightly. In the current study, some of the indicators were redefined based on recent research using Morimoto’s HPI [30], the leading health indicators of Health Plan 2020 [31], and the legal working hours in South Korea that changed in 2018 [32]. In the current study, the operationalized definitions of positive health behaviors on the HPI were:

1. Amount of Sleep: Sleeping 7 to 8 h/day;
2. Eating breakfast: Eating breakfast 5 to 7 times/week;
3. Mental stress: Not feeling much stress in everyday life;
4. Smoking: Non-smoker (currently non-smoking and less than 5 packs for a lifetime);
5. Alcohol use: Not being a high-risk drinker (i.e., men who drink seven or more alcoholic drinks on average per occasion and women who drink five or more alcoholic drinks on average per occasion, at least twice a week);
6. Physical activity: Engaging in aerobic physical activity (i.e., >2 h and 30 min of moderate-intensity physical activity per week; >1 h and 15 min of high-intensity physical activity per week; or mixed participation in moderate- and high-intensity physical activities for an equivalent time for each activity);
7. Nutritionally balanced diet: Practicing two or more of the following dietary behaviors: (1) Having a fat intake of 15–25%; (2) Having a daily sodium intake of 2000 mg or less; (3) Having a daily vegetable and fruit intake of 500 g or more; (4) Reading nutrition labels;
8. Working hours: Working ≤52 h/week.

#### 2.1.2. MetS

Using the diagnostic criteria of the National Cholesterol Education Program Adult Treatment Panel III [9], MetS was defined as the presence of three or more of the following five indicators: (1) Elevated waist circumference (i.e., ≥90 cm in men; ≥85 cm in women); (2) Elevated fasting glucose (i.e., ≥100 mg/dL) or taking medication for elevated blood glucose; (3) Elevated triglycerides (i.e., ≥150 mg/dL) or taking medication for elevated triglycerides; (4) Reduced high-density lipoprotein cholesterol (HDL-C) (i.e., <40 mg/dL in men; <50 mg/dL in women) or taking medication for low HDL-C; (5) Elevated blood pressure (i.e., ≥130/85 mmHg) or taking medication for hypertension. The waist circumference cut-off points for central obesity in Korean adults were determined using the criteria determined by the Korean Society for the Study of Obesity [33].

#### 2.1.3. Covariates

The covariates that were found to be associated with MetS in previous research were included in the analysis. They were age [6,7,34,35,36,37], socioeconomic status [36,38,39], education level [36,38], gender [34,36,37,40], being in menopause for women [36], and household status [35,38,41].

### 2.2. Ethical Considerations

As the primary study, the KNHANES, a national survey and designated statistics, was conducted with the approval of the Institutional Review Board of the Korea Disease Control and Prevention Agency. The raw data from this study were processed to safeguard the individual identity of the survey participants according to the Personal Information Protection Act and the Statistics Act and were downloaded from the website of the Korea National Health and Nutrition Examination Survey (https://knhanes.cdc.go.kr/knhanes/main.do, accessed on 15 September 2020) after agreeing to the user compliance pledge for the statistical data regarding prohibitions for using the data for purposes other than research or without maintaining confidentiality and registering the user information. The data were used after carefully reading the guidelines regarding the use of the raw data of the Seventh KNHANES [42].

### 2.3. Statistical Analyses

Statistical analyses were performed using SAS version 9.4 (SAS Institute Inc., Cary, NC, USA) and the statistical program R, version 4.0.2 (http://cran.r-project.org, accessed on 15 September 2020). Statistical significance was set at *p* < 0.05, and a 95% confidence interval was employed. We analyzed the KNHANES data with primary sampling units, strata, and integrated sample weights, constructed for sample participants considering the Korean population regarding complex survey design, survey non-response, and post-stratification. Differences in MetS by the covariates were analyzed using the independent *t*-test for continuous variables and the weight-applied Rao–Scott chi-square test for categorical variables. Complex sample multiple logistic regression analysis was conducted to determine associations between the HPI and MetS after adjusting for age, household, menopause (only for women), household income, and education level.

## 3. Results

Gender differences in demographic characteristics for the risk of MetS are shown in Table 1. Among the sample of 8677 middle-aged adults (men: *n* = 3813; women: *n* = 4864), 2211 individuals (25.5%) had MetS (men: *n* = 1238; women: *n* = 973). The mean age of the participants with MetS was 53.01 years (SD = 6.78). Among the individuals with MetS, 8.0% (*n* = 99) of the men and 10.8% (*n* = 105) of the women were living in single households, and 70.5% of (*n* = 639) the women with MetS had menopause. For the overall sample, the result of the Rao–Scott chi-square test revealed that the risk of MetS significantly differed by age (*p* < 0.001), gender (*p* < 0.001), household income (*p* < 0.001), education level (*p* < 0.001), smoking (*p* < 0.001), alcohol use (*p* < 0.001), physical activity (*p* < 0.001), mental stress (*p* = 0.027), having a nutritionally balanced diet (*p* < 0.001), and HPI score (*p* < 0.001). However, there were factors associated with the risk of MetS that differed by gender. Smoking in men was a significant factor for the risk of MetS, while living arrangement, menopause, education level, working hours, and balanced diet were related to the risk of MetS among women.

Table 2 shows the gender differences in HPI characteristics for individuals with MetS. There were significant gender differences in smoking, alcohol use, working hours, and having a nutritionally balanced diet for respondents with MetS. Compared with women, men with MetS were more likely to smoke 5 packs or more (*p* < 0.001), engage in high-risk alcohol use (*p* < 0.001), work less than 52 h/week (*p* < 0.001), and not have a nutritionally balanced diet (*p* < 0.001). There were no gender differences in eating breakfast, amount of sleep, physical activity, and mental stress.

### 3.1. Demographic Characteristics and Total HPI Score Associated with MetS by Gender

Table 3 shows the demographic characteristics and total HPI scores that were associated with the risk of MetS by gender. For both men and women, age (*p* < 0.001), gender (*p* < 0.001), household income (*p* < 0.001 for low income; *p* = 0.019 for middle income), educational level (*p* = 0.007 for below middle school education; *p* = 0.034 for high school education), and total HPI score (*p* = 0.003 for poor; *p* = 0.024 for moderate) were statistically significant in association with the risk for MetS. For men, age and total HPI score were significantly associated with MetS. Older men were 1.027 times more likely to have a risk for MetS. In addition, men with poor HPI scores and those with moderate HPI scores were, respectively, 1.486 and 1.544 times more likely to have a risk for MetS than men with good HPI scores. For women, age, household income, educational level, and total HPI scores were significantly related to the risk for MetS. Older women were 1.028 times more likely to have a risk for MetS. Women with less than middle school education and those with a high school education were, respectively, 2.033 and 1.585 times more likely to have a risk for MetS compared with those with a university education or above. In addition, compared with those with good HPI scores, women with poor HPI scores were 1.962 times more likely to have a risk for MetS, while those with moderate HPI scores were 1.356 times more likely to have a risk for MetS.

### 3.2. General Characteristics and Each Indicator of HPI Associated with the Risk of MetS by Gender

Table 4 shows the demographic characteristics and each indicator of HPI related to the risk for MetS by gender. For men and women, age (*p* < 0.001), gender (*p* < 0.001), household income (low: *p* < 0.001, middle: *p* = 0.017), education level (below middle school; *p* = 0.013), alcohol use (*p* < 0.001), and physical activity (*p* = 0.008) were significantly associated with the risk for MetS. There were gender differences for the demographic characteristics and the HPI indicators for the risk of MetS. For men, age (*p* < 0.001), household (*p* = 0.045), household income (*p* = 0.011 for low), alcohol use (*p* < 0.001), and physical activity (*p* = 0.026) were significantly related to the risk for MetS. For women, household income (*p* = 0.004 for low), educational level (*p* < 0.001 for both below middle school and high school education), physical activity (*p* = 0.047), and having a nutritionally balanced diet (*p* = 0.007) were significantly associated with the risk of MetS. After controlling for the demographic covariates, men with high-risk alcohol use were 2.056 times more likely, and those who were not physically active were 1.250 times more likely to have a risk for MetS. After adjusting for demographic covariates, women who were not physically active were 1.203 times more likely, and those not having a nutritionally balanced diet were 1.306 times more likely to have a risk for MetS. 

## 4. Discussion

As the prevalence of MetS increases by age, middle-aged adults are at a critical period for preventing and managing MetS to improve health in old age. Modifying unhealthy behaviors is a crucial method for preventing and managing MetS. Since health behaviors are known to differ by gender, it is necessary to identify the influence of gender on lifestyle habits regarding the risk of MetS. Using the HPI, we found commonalities and differences in health behaviors by gender for the risk of MetS.

Compared with those with poor and moderate HPI scores, individuals with good HPI scores had a lower risk for MetS in both middle-aged men and women. This finding is inconsistent with another study that analyzed the association between Morimoto’s HPI and the 7-year incidence rate of MetS [4], which found no differences in the 7-year incidence rate of MetS between the good and moderate groups for women but significant differences in the 7-year incidence rate between the poor and moderate groups for men. This disagreement may be due to differences in the operational definitions used to measure health behaviors on the HPI. In the current study, compared with previous research, we used more specific, comprehensive, and objective definitions of health behaviors and found a clearer association with MetS in middle-aged men and women. For example, Morimoto’s HPI only presented the frequency of alcohol use. However, we utilized the variable of high-risk alcohol use that considered both the frequency and amount of alcohol consumption since an appropriate level of alcohol consumption, such as a light drink, can have a positive effect on MetS [43]. Thus, it may be both practical and useful to define alcohol use as high-risk drinking, as was done in this study. In addition, Morimoto’s HPI presented only the frequency of physical activity without considering the intensity, but our modified index specified aerobic physical activity calculated in terms of frequency, intensity, and time in both leisure activities and daily life. Since the effects of low-intensity physical activity in preventing MetS have not been clearly determined [44], it may be more appropriate to use the HPI with the indicator modified to assess moderate- to high-intensity physical activities in MetS management.

Morimoto’s HPI consists of subjective questions about a nutritionally balanced diet. However, numerous studies [15,19,23,25,27,45,46,47] have indicated that various dietary habits, including a Mediterranean diet, nut intake, and animal by-product food intake, are associated with MetS. Thus, it is necessary to define the healthy eating habits associated with MetS more objectively and specifically than is currently being done. The healthy diet indicators modified for this study, including a low-sodium diet, high vegetable and fruit intake, a low-fat diet, and increased interest in nutritional values, were similar to the guidelines for the DASH (Dietary Approaches to Stop Hypertension)-Sodium diet (e.g., rich in fruits, vegetables, low-fat, and low sodium dairy product). Such indicators are also consistent with low-fat, low-sodium intake and increased vegetable and fruit intake diets presented by the American Heart Association Diet and Lifestyle Recommendations [12]. In previous studies [15,19,23,45,46,47], the intake of vegetables and fruits, sources of potassium, magnesium, and fiber [45], as well as a low-sodium diet and a low-fat diet have been reported to have positive effects on high blood pressure, triglycerides, and HDL-C, thereby reducing the risk for MetS. Therefore, the modified Morimoto’s HPI used in this study may effectively manage the lifestyle risk factors for the prevention of MetS in middle-aged adults.

In this study, we found that moderate- to high-intensity physical activity was an important lifestyle factor in reducing the risk for MetS in both middle-aged men and women. These findings are consistent with the results of previous studies suggesting that moderate- to high-intensity physical activity prevents and improves MetS [44,48]. Physical activity is effective in reducing central obesity [10,49], the most common diagnostic component of MetS [15,48]. However, the independent effects of physical activity on the other diagnostic criteria of MetS have not yet been clearly identified, although it has been shown to increase energy metabolism by increasing muscle and to have a positive effect on insulin resistance and lipid metabolism [5]. Therefore, physical activity appears to be a critical lifestyle habit that should be emphasized for preventing MetS among middle-aged men and women. However, the practice of moderate- to high-intensity physical activity has been reported to be declining in South Korea (i.e., 54.7% of women and 62.0% of men in 2013 to 44.0% of women and 51% of men in 2018) [50]. Therefore, health promotion programs should be developed to increase the levels of physical activity for middle-aged adults.

In the current study, high-risk alcohol use was the lifestyle factor most strongly associated with the increased risk for MetS, which is consistent with previous research [43]. Alcohol consumption was associated with elevated waist circumference, fasting serum glucose, blood pressure, and triglycerides [51,52,53]. In particular, high-risk alcohol use has been shown to influence obesity, which contributes to increases in waist circumference, the most common diagnostic component for MetS [52,53,54].

Gender differences in the association between high-risk alcohol use and MetS were found. High-risk drinking was the strongest risk factor for MetS among middle-aged men but not middle-aged women, consistent with a study that systematically reviewed alcohol consumption and risk for MetS [43]. Gender differences in high-risk alcohol use and MetS might be explained by the gender-specific diagnostic components of MetS. Alcohol consumption aggravates hypertension, triacylglycerol levels, and hyperglycemia [51,52,53]. Among the diagnostic components of MetS, abnormal triacylglycerol levels and hyperglycemia have been reported to be more frequently found in men than in women [10,26,49]. Thus, high-risk alcohol use could be a lifestyle habit that influences abnormal triacylglycerol and plasma glucose levels, which are the key indicators for MetS in men. However, the positive effect of alcohol consumption is that it increases HDL-C levels [52,53]. Among the diagnostic components of MetS, low levels of HDL-C have been more frequently found in women than in men [10,49], which may indicate that alcohol use among women may play a positive effect. Consequently, alcohol drinking in men could strengthen health indicators related to the diagnostic components of MetS while weakening them in women.

The differences in the frequency of high-risk drinking habits among men and women may have also contributed to gender differences in the association between high-risk alcohol use and MetS. In this study, compared with the 18.8% of middle-aged men who engaged in high-risk alcohol use, only 3.1% of the middle-aged women with MetS did, indicating a sizable difference in the frequency of high-risk drinking between men and women. Such results highlight the necessity of operating a MetS management program for adults who engage in high-risk drinking that focuses on middle-aged men, who are more likely to engage in high-risk alcohol use and for whom high-risk drinking is the most important risk factor, rather than for middle-aged women who have lower rates of high-risk drinking.

Having a nutritionally balanced diet was found to be a lifestyle factor associated with the prevention of MetS in middle-aged women; however, it was not a significant factor for middle-aged men. A previous study that analyzed the dietary patterns of South Koreans [27] identified an association between diet and MetS for women but not men. In a study that systematically reviewed the association between a healthy diet and MetS [19], healthy eating behaviors were associated with MetS in both men and women but had a stronger protective effect for women than for men. The results showed that meat-based and fried food diets increased the risk of MetS only in men, and high-salt and high-calorie diets increased the risk of MetS only in women [25]. As such, differences in the association between diet and MetS by gender have not been clearly explained [27]. Sex hormones (i.e., estrogen and testosterone) may affect triacylglycerol and HDL-C, which are diagnostic components of MetS [27]. Further, differences in dietary patterns by gender have also been reported to influence the pathological development of MetS [25]. In this study, differences in healthy eating practices were identified by gender. Since in this study having a nutritionally balanced diet was identified as the most effective indicator of the lifestyle habits for middle-aged women, it would be beneficial to emphasize the importance of maintaining a nutritionally balanced diet in a MetS management program for middle-aged women.

The prevalence of MetS was higher in the middle-aged men than in the middle-aged women in this study, which was similar to the results of recent previous studies that reported the prevalence of MetS in adults in the United States, Korea, and Taiwan [6,7,10]. Unhealthy lifestyle habits can explain the high prevalence of MetS in middle-aged men compared with middle-aged women [10,54]. Differences in lifestyle practices between men and women were also identified in this study. However, in this study, the prevalence of MetS was still high in men even after controlling for the lifestyle habits associated with the syndrome. The higher prevalence of MetS in men than in women has also been explained by the higher level of hormones, including androgen in men, which affects the pathologies triggering MetS, such as insulin resistance, increase in belly fat, and hypertension [55]. The sex-specific cut-off levels, such as the ones for HDL-C and central obesity, have sometimes been suggested as reasons for the differences in MetS between men and women [55]. A more active MetS prevention program tailored to men who are at risk for developing unhealthy lifestyle habits would be beneficial.

### 4.1. Limitations

This study aimed to identify how the associations between MetS by lifestyle indicators on the HPI might be modified by gender. However, as a cross-sectional study, this study cannot suggest causal relationships between the modified HPI and MetS. In addition, the results of this research are relative to the Korean population; it is difficult to generalize the findings to other areas.

### 4.2. Future Directions

To determine the causal relationships between the modified HPI and the risk for MetS, a longitudinal study would be required. In this study, associations of the total score of the modified HPI, physical activity, high-risk drinking, and nutritionally balanced diets with MetS were identified. However, some indicators of HPI, such as the amount of sleep, eating breakfast, working hours, and mental stress were not found to be significantly associated. Further research is required to explore the association between MetS and the lifestyle habits that were not identified in this study as being statistically significant. Moreover, the development of a modified HPI suitable for the prevention and management of MetS would be required to compare the association between the total HPI score and MetS after modifying or deleting irrelevant areas from the HPI.

## 5. Conclusions

In this study, the middle-aged men and women categorized as being in the good HPI group, with a total score of 6 or higher on the modified HPI, had a lower risk of MetS than those in the poor and moderate groups. These results could be used to present target scores for lifestyle habits that need to be improved through lifestyle improvement programs to prevent and manage MetS in middle-aged adults. In this study, there were differences in the associations between lifestyle habits and MetS by gender. Physical activity was associated with MetS in both men and women. High-risk drinking behavior was the most important lifestyle habit for men while having a nutritionally balanced diet was the most significant lifestyle habit for women. These findings suggest that gender is a crucial factor that should be considered in interventions for the prevention and management of MetS. The importance of refraining from high-risk alcohol use and increasing physical activity should be emphasized in interventions for the prevention and management of MetS that target middle-aged men, whereas programs that focus on physical activity and diet would be beneficial for middle-aged women.

## Figures and Tables

**Table 1:** Demographic characteristic of the risk of metabolic syndrome (MetS) by gender (*n* =8677).

Characteristics	All				Men				Women			
	MetS				MetS				MetS			
	No (*n* = 6466)	Yes (*n* = 2211)	χ^2^	*p*	No (*n* = 2575)	Yes (*n* = 1238)	χ^2^	*p*	No (*n* = 3891)	Yes (*n* = 973)	χ^2^	*p*
	*n* (%) or *M* (*SD*)	*n* (%) or *M* (*SD*)			*n* (%) or *M* (*SD*)	*n* (%) or *M* (*SD*)			*n* (%) or *M* (*SD*)	*n* (%) or *M* (*SD*)		
Age	51.13 (6.89)	53.01 (6.78)	8.91	<0.001	51.21 (6.94)	52.2 (6.87)	3.61	<0.001	51.07 (6.86)	54.03 (6.53)	10.89	<0.001
Gender												
Men	2575 (39.8)	1238 (56.0)	202.67	<0.001								
Women	3891 (60.2)	973 (44.0)										
Living arrangement												
Not living alone	5973 (92.4)	2007 (90.8)	1.30	0.297	2333 (90.6)	1139 (92.0)	3.22	.103	3640 (93.5)	868 (89.2)	14.18	<0.001
Living alone	493 (7.6)	204 (9.2)			242 (9.4)	99 (8.0)			251 (6.5)	105 (10.8)		
Menopause												
Yes									1813 (51.5)	639 (70.5)	119.51	<0.001
No									1708 (48.5)	267 (29.5)		
Household income												
Low	1532 (23.7)	639 (28.9)	53.81	<0.001	633 (24.6)	323 (26.1)	13.30	0.004	899 (23.1)	316 (32.5)	55.45	<0.001
Middle	3227 (49.9)	1112 (50.3)			1288 (50.0)	624 (50.4)			1939 (49.8)	488 (50.2)		
High	1707 (26.4)	460 (20.8)			654 (25.4)	291 (23.5)			1053 (27.1)	169 (17.4)		
Education level												
Below middle school	1889 (29.2)	737 (33.3)	45.68	<0.001	788 (30.6)	303 (24.5)	3.43	0.250	1101 (28.3)	434 (44.6)	150.04	<0.001
High school	2188 (33.8)	799 (36.1)			736 (28.6)	433 (35.0)			1452 (37.3)	366 (37.6)		
Greater than university	2389 (36.9)	675 (30.5)			1051 (40.8)	502 (40.5)			1338 (34.4)	173 (17.8)		
Health Practice Index												
Smoking												
5 packs or more	1058 (17.5)	627 (28.7)	97.51	<0.001	882 (37.8)	554 (45.3)	12.80	0.001	176 (4.8)	73 (7.6)	3.33	0.107
Not *	4977 (82.5)	1559 (71.3)			1449 (62.2)	669 (54.7)			3528 (95.2)	890 (92.4)		
Alcohol use												
High-risk drinking	667 (11.0)	479 (21.9)	184.67	<0.001	487 (20.9)	412 (33.7)	79.1	<0.001	180 (4.9)	67 (7.0)	6.76	0.026
Not *	5373 (89.0)	1707 (78.1)			1847 (79.1)	811 (66.3)			3526 (95.1)	896 (93.0)		
Eating breakfast												
Irregularly or never	3841 (68.4)	1280 (69.2)	0.01	0.923	1448 (68.5)	685 (68.6)	0.68	0.507	2393(68.3)	595(69.9)	0.52	0.551
Almost daily *	1776 (31.6)	570 (30.8)			666 (31.5)	314 (31.4)			1110(31.7)	256(30.1)		
Amount of sleep												
<7 h/day or >8 h/day	3970 (66.1)	1494 (68.5)	1.81	0.242	1527 (65.9)	808 (66.3)	0.09	0.784	2443 (66.2)	686 (71.2)	4.77	0.056
7–8 h/day *	2036 (33.9)	687 (31.5)			791 (34.1)	410 (33.7)			1245 (33.8)	277 (28.8)		
Working hours												
Over 52 h/week	2514 (41.2)	954 (43.1)	1.88	0.230	874 (36.9)	466 (37.6)	0.80	0.423	1640 (43.9)	488 (50.2)	10.18	0.005
Less than 52 h/week *	3594 (58.8)	1257 (56.9)			1494 (63.1)	772 (62.4)			2100 (56.1)	485 (49.8)		
Physical activity												
Not	3143 (54.5)	1319 (62.7)	30.21	<0.001	1157 (52.8)	716 (61.0)	18.51	<0.001	1986 (55.5)	603 (64.8)	15.25	<0.001
Aerobic physical activity *	2626 (45.5)	786 (37.3)			1035 (47.2)	458 (39.0)			1591 (44.5)	328 (35.2)		
Mental Stress												
High	1486 (24.3)	607 (27.5)	6.22	0.027	562 (23.7)	338 (27.3)	4.31	0.059	924 (24.7)	269 (27.6)	2.35	0.175
Moderate or low *	4622 (75.7)	1604 (72.5)			1806 (76.3)	900 (72.7)			2816 (75.3)	704 (72.4)		
Having a nutritionally balanced Diet												
No	2684 (41.5)	1102 (49.8)	33.91	<0.001	1351 (52.5)	656 (53.0)	0.00	0.979	1333 (34.3)	446 (45.8)	35.63	<0.001
Yes *	3782 (58.5)	1109 (50.2)			1224 (47.5)	582 (47.0)			2558 (65.7)	527 (54.2)		
Health Practice Index Score **												
Poor	505 (10.1)	322 (18.3)	128.02	<0.001	312 (17.4)	213 (22.5)	31.28	<0.001	193 (6.0)	109 (13.4)	58.94	<0.001
Moderate	815 (16.3)	365 (20.7)			337 (18.8)	211 (22.3)			478 (14.9)	154 (18.9)		
Good	3684 (73.6)	1075 (61.0)			1140 (63.7)	522 (55.2)			2544 (79.1)	553 (67.8)		

*Note*: *M* mean; *SD* standard deviation; * Positive lifestyle (1 point). ** Health Practice Index score: The sum of each positive health behavior: Poor (0–3 points), Moderate (4–5 points), Good (6–8 points).

**Table 2:** Gender differences in Health Practice Index (HPI) characteristics for the individuals with MetS (*n* = 2211).

Health Practice Index	MetS			
	Men (*n* = 1238)	Women (*n* = 973)	χ^2^	*p*-Value
	*n* (%) or *M* (*SD*)	*n* (%) or *M* (*SD*)		
Smoking				
5 packs or more	554 (25.3)	73 (3.3)	337.28	<0.001
Not *	669 (30.6)	890 (40.7)		
Alcohol use				
High-risk alcohol use	412 (18.8)	67 (3.1)	202.47	<0.001
Not *	811 (37.1)	896 (41.0)		
Eating breakfast				
Irregularly or never	685 (37.0)	595 (32.2)	0.50	0.561
Almost daily *	314 (17.0)	256 (13.8)		
Amount of sleep				
<7 h/day or >8 h/day	808 (37.0)	686 (31.5)	3.77	0.081
7–8 h/day *	410 (18.8)	277 (12.7)		
Working hours				
Over 52 h/week	466 (21.1)	488 (22.1)	32.17	<0.001
Less than 52 h/week *	772 (34.9)	485 (21.9)		
Physical activity				
Not	716 (34.0)	603 (28.6)	1.80	0.236
Aerobic physical activity *	458 (21.8)	328 (15.6)		
Mental stress				
High	338 (15.3)	269 (12.2)	0.06	0.820
Moderate or low *	900 (40.7)	704 (31.8)		
Having a nutritionally balanced diet				
No	656 (29.7)	446 (20.2)	13.37	<0.001
Yes *	582 (26.3)	527 (23.8)		

*Note*: *M* mean; *SD* standard deviation * Positive lifestyle (1 point).

**Table 3:** Demographic characteristics and total HPI score associated with the risk of MetS.

Characteristics	All					Men					Women				
	*B*	*SE*	Adjusted Odds Ratio (95% CI)		*p*	*B*	*SE*	Adjusted Odds Ratio (95% CI)		*p*	*B*	*SE*	Adjusted Odds Ratio (95% CI)		*p*
Age	0.033	0.005	1.033 (1.023,1.044)		<0.001	0.026	0.007	1.027 (1.013, 1.040)		<0.001	0.028	0.014	1.028 (1.001, 1.056) *		0.044
Gender															
Men	0.714	0.066	2.041 (1.793, 2.324)		<0.001										
Women	Ref														
Living Arrangement															
Not living alone	0.127	0.118	1.136 (0.900, 1.433)		0.283	0.285	0.170	1.330 (0.951,1.858)		0.095	−0.133	0.159	0.876 (0.641, 1.196)		0.403
Living alone	Ref					Ref					Ref				
Menopause															
Yes											0.319	0.174	1.388 (0.986, 1.952)		0.060
No											Ref				
Household income															
Low	0.380	0.095	1.462 (1.213, 1.762)		<0.001	0.274	0.130	1.315 (1.018, 1.698)		0.036	0.427	0.145	1.532 (1.152, 2.037)		0.003
Middle	0.199	0.085	1.22 (1.033, 1.441)		0.019	0.165	0.117	1.179 (0.937, 1.484)		0.159	0.210	0.128	1.234 (0.959, 1.586)		0.101
High	Ref					Ref					Ref				
Education Level															
Below Middle school	0.266	0.098	1.305 (1.076, 1.582)		0.007	−0.051	0.140	0.95 (0.722, 1.250)		0.715	0.709	0.153	2.033 (1.504, 2.747)		<0.001
High school	0.171	0.081	1.187 (1.013, 1.391)		0.034	0.042	0.108	1.043 (0.844, 1.290)		0.696	0.461	0.129	1.585 (1.232, 2.041)		<0.001
Greater than University	Ref					Ref					Ref				
HPI score															
Poor	0.309	0.104	1.363 (1.111, 1.671)		0.003	0.396	0.123	1.486 (1.166, 1.894)		0.001	0.674	0.156	1.962 (1.443, 2.667)		<0.001
Moderate	−0.180	0.080	0.835 (0.714, 0.977)		0.024	0.434	0.111	1.544 (1.241, 1.920)		<0.001	0.304	0.117	1.356 (1.076, 1.708)		0.010
Good	Ref					Ref					Ref				
	Likelihood Ratio *F* = 40.13 (8.81, 4597.49) *p* < 0.001, Max-rescaled *R*^2^ = 0.077					Likelihood Ratio *F* = 6.31 (7.81, 4074.69) *p* < 0.001, Max-rescaled *R*^2^ = 0.025					Likelihood Ratio *F* = 24.42 (8.83, 4607.05) *p* < 0.001, Max-rescaled *R*^2^ = 0.085				

*Note: B* standardized regression coefficient; *SE* Standard Error; Ref Reference group. * Positive lifestyle (1 point).

**Table 4:** General characteristics and each indicator of HPI related to the risk of MetS by gender (*n* = 8677).

Characteristics	All				Men				Women			
	*B*	*SE*	Adjusted Odds Ratio (95% CI)	*p*	*B*	*SE*	Adjusted Odds Ratio (95% CI)	*p*	*B*	*SE*	Adjusted Odds Ratio (95% CI)	*p*
Demographics												
Age	0.039	0.006	1.040 (1.028, 1.051)	<0.001	0.029	0.007	1.029 (1.015, 1.044)	<0.001	0.026	0.014	1.027 (0.999, 1.055)	0.060
Gender												
Men	0.621	0.075	1.861 (1.605, 2.158)	<0.001								
Women	Ref											
Living arrangement												
Not living alone	0.177	0.118	1.193 (0.947, 1.504)	0.135	0.347	0.173	1.415 (1.008, 1.986)	0.045	−0.11	0.157	0.895 (0.657, 1.218)	0.479
Living alone	Ref				Ref				Ref			
Menopause												
Yes									0.333	0.175	1.395 (0.989, 1.968)	0.058
No									Ref			
Household income												
Low	0.386	0.096	1.472 (1.218, 1.778)	<0.001	0.332	0.131	1.394 (1.078, 1.801)	0.011	0.424	0.146	1.529 (1.147, 2.038)	0.004
Middle	0.205	0.085	1.227 (1.038, 1.451)	0.017	0.195	0.117	1.215 (0.965, 1.53)	0.098	0.211	0.128	1.235 (0.960, 1.590)	0.101
High	Ref				Ref				Ref			
Education level												
Below middle school	0.244	0.098	1.277 (1.053, 1.548)	0.013	−0.07	0.141	0.934 (0.709, 1.232)	0.630	0.682	0.154	1.979 (1.462, 2.678)	<0.001
High school	0.141	0.081	1.151 (0.982, 1.349)	0.082	0.003	0.11	1.003 (0.807, 1.246)	0.981	0.459	0.128	1.582 (1.230, 2.036)	<0.001
Greater than university	Ref				Ref				Ref			
Health Practice Index												
Smoking												
5 packs or more	0.127	0.091	1.135 (0.950, 1.357)	0.164	0.134	0.098	1.143 (0.943, 1.385)	0.172	0.123	0.223	1.131 (0.730, 1.753)	0.580
Not *	Ref				Ref				Ref			
Alcohol use												
High risk drinking	0.643	0.094	1.902 (1.581, 2.288)	<0.001	0.721	0.102	2.056 (1.681, 2.514)	<0.001	0.346	0.234	1.414 (0.893, 2.238)	0.139
Not *					Ref				Ref			
Eating breakfast												
Irregularly or never	−0.1	0.076	0.909 (0.783, 1.055)	0.208	−0.1	0.108	0.903 (0.730, 1.117)	0.347	−0.09	0.107	0.917 (0.742, 1.132)	0.418
Almost daily *	Ref				Ref				Ref			
Amount of sleep												
<7 h/day or >8 h/day	0.079	0.072	1.082 (0.940, 1.246)	0.272	0.038	0.091	1.039 (0.869, 1.241)	0.676	0.125	0.104	1.133 (0.923, 1.390)	0.233
7–8 h/day *	Ref				Ref				Ref			
Working hours												
Over 52 h/week	0.079	0.069	1.082 (0.946, 1.238)	0.249	0.061	0.101	1.063 (0.872, 1.295)	0.547	0.119	0.094	1.127 (0.937, 1.355)	0.204
Less than 52 h/week *	Ref				Ref				Ref			
Physical activity												
Not	0.178	0.067	1.195(1.047, 1.364)	0.008	0.223	0.1	1.250 (1.027, 1.521)	0.026	0.185	0.093	1.203 (1.002, 1.443)	0.047
Aerobic physical activity *	Ref				Ref				Ref			
Mental stress												
High	0.104	0.076	1.109 (0.955, 1.289)	0.175	0.132	0.105	1.141 (0.928, 1.403)	0.212	0.093	0.103	1.098 (0.897, 1.343)	0.364
Moderate or low *	Ref				Ref				Ref			
Having a nutritionally balanced diet												
No	0.035	0.068	1.036 (0.906, 1.184)	0.606	−0.13	0.093	0.876 (0.730, 1.051)	0.154	0.267	0.099	1.306 (1.075, 1.587)	0.007
Yes *	Ref				Ref				Ref			
	Likelihood Ratio *F* = 28.19 (14.45, 7542.94) *p* < 0.001, Max-rescaled *R2* = 0.090				Likelihood Ratio *F* = 7.72 (13.58, 7087) *p* < 0.001, Max-rescaled *R2* = 0.054				Likelihood Ratio *F* = 14.55 (14.54, 7590.79) *p* < 0.001, Max-rescaled *R2* = 0.086			

*Note*. *B* standardized regression coefficient; *SE* Standard Error; CI Confidence Interval; Ref Reference group; * Positive lifestyle (1 point).

## Data Availability

Publicly available datasets were analyzed in this study.
